# Author Correction: An ecological network approach to predict ecosystem service vulnerability to species losses

**DOI:** 10.1038/s41467-021-26195-x

**Published:** 2021-09-30

**Authors:** Aislyn A. Keyes, John P. McLaughlin, Allison K. Barner, Laura E. Dee

**Affiliations:** 1 Department of Ecology and Evolutionary Biology, University of Colorado, Boulder, CO USA; 2 Depeartment of Ecology, Evolution, and Marine Biology, University of California, Santa Barbara, CA USA; 3 Department of Biology, Colby College, Waterville, ME USA

**Keywords:** Food webs, Ecological modelling, Ecosystem services

Correction to: *Nature Communications* 10.1038/s41467-021-21824-x, published online 11 March 2021.

In the original version of the published article, two formulas in the “Robustness analysis” section of the Methods were incorrectly expressed as $R_F=\sum x(y)$ and $R_{ES}=\sum x(y)$. The formulas have now been correctly expressed as ${R}_{F}=\frac{\sum y(x)}{max(x)}$ and ${R}_{ES}=\frac{\sum y(x)}{max(x)}$.

In addition, the following sentence was added to the second paragraph of the “Robustness analysis” section for clarity: “*Our species loss sequences did not all include the full species list, so we divided the AUC by the maximum proportion of species removed to scale robustness to the length of the sequence.*”. This has now been corrected in both the PDF and HTML versions of the Article.

