# Supplementary material for: An ecological network approach to predict ecosystem service vulnerability to species losses
Source: Nat Commun. 2021 Mar 11;12:1586. doi: 10.1038/s41467-021-21824-x (PMC7952599; doi:10.1038/s41467-021-21824-x)
Supplement: Supplementary file 1 — Supplementary Information [file 41467_2021_21824_MOESM1_ESM.pdf]

Supplementary Information for
An ecological network approach to predict ecosystem service vulnerability to species losses
Authors: Keyes, McLaughlin, Barner and Dee

Table of Contents

I. Supplementary Methods **2**
Figure 1: Locations of the three salt marsh datasets 2
I.1 Ecosystem service (ES) assignment to species 2
Table 1: Number of spp. providing ES (i.e., redundancy) 3
I.2 Species loss sequences 3
Figure 2: Robustness for extinction sequences of ecosystem service providers 4
Figure 3: Conceptual Personalized PageRank example 5
Figure 4: Robustness for 3 different alpha values (supporting spp.) 6
I.3 Robustness methods and sensitivity analyses 8
Figure 5: Illustration of robustness calculations 9
I.4 Robustness of ecosystem services with weighted species contributions 10
Figure 6: Illustration of weighted robustness calculations 11
II. Supplementary Discussion **12**
Figure 7: Food web and ES responses to primary removals and secondary losses 12
Figure 8: Aggregate ecosystem service robustness is generally lower when weights are included 13
Figure 9: Food web and weighted ecosystem service robustness remain correlated 14
II.1 Correlation not skewed by the length of the vulnerable species sequence 14
II.2 Ecosystem service robustness across systems and sequences 15
II.3 Food web robustness across sequences 15
II.4 Robustness to short sequences 15
Table 2: Length of species loss sequences. 15
Table 3: Number of secondary losses across sequences 16
II.5 Is ecosystem service robustness a function of sample size? 17
Table 4: Selecting species that match ES in trophic level and in-degree centrality 17
Table 5: Comparing the robustness of ES and species with similar trophic level and centrality 18
Table 6: Tracking when ecosystem services and matching species are secondarily lost 18
Figure 10: Spp. losses causing the secondary loss of ES and spp. similar to ES 19
II.6 Sensitivity of results to the exclusion of parasites 20
Table 7: Spp. losses causing the secondary loss of ES and spp. similar to ES 20
Figure 11: Food web and ecosystem service robustness remain correlated when parasites are included 21
Figure 12: Robustness values generally increase when parasites are included versus excluded 21
II.7 Individual ecosystem service robustness is driven by trophic level and redundancy 22
Table 8: Regression results: ES robustness as a function of trophic level and redundancy 22
III. Supplementary References **23**

I. Supplementary Methods

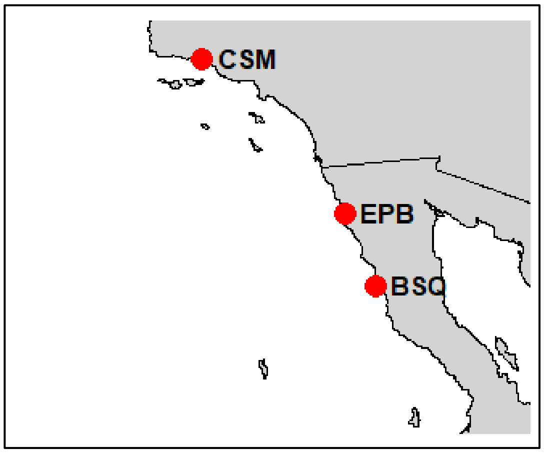

Figure 1. **Locations of the three salt marsh datasets.** CSM: Carpinteria Salt Marsh, California, USA. EPB: Estero de Punta Banda, Baja, Mexico. BSQ: Baha Falsa de San Quintin, Baja Mexico. Food web data come from Hechinger et *al.*[1]

I.1 Ecosystem Service Assignment to Species

We assigned ecosystem services to species after filtering the original salt marsh food webs[1] to exclude parasites and detritus, and to only include adult life stages. Then, we conducted a literature search in Google Scholar to select the main ecosystem services provided by salt marsh ecosystems: shoreline protection, wave attenuation, water filtration, carbon sequestration, fisheries, birdwatching and waterfowl hunting. To determine ecosystem service providers for shoreline protection, wave attenuation, water filtration, carbon sequestration, we conducted a literature search in Google Scholar, following previous methods[2]. Specifically, we searched for each unique service – species combination, using both species common and scientific names, as well as synonyms for each ecosystem service. For example, carbon sequestration could also be published as "carbon storage." If a reference existed that indicated a species provided a service, we augmented the interaction data (link data) to include a link from the species to the service. For the fishery and waterfowl ecosystem services we referenced government documents with seasonal regulations and/or catch limits and guidelines (e.g. [3,4]). Data will be published for the ecosystem service network layer (Dee and Keyes, in review).

Birdwatching. We used the R package auk v 0.4.1 to process and analyze the data[5]. We filtered the eBird Basic Dataset by Complete Checklists and by spatial extent matching the eBird registered hotspots with the latitude and longitude from the original food web data[1]. We did not filter by month or year, instead using all records from 1900-2019. For each estuary, we aggregated count data by species to get a total count (i.e., total number of birds seen per species across all complete checklists) which we used to calculate the relative frequency at each estuary. Species with lower relative frequencies were referred to as rarer than those with higher relative frequencies.

Waterfowl Hunting. For Carpinteria Salt Marsh, we assigned links for bird species that are hunted and regulated in the Southern California Zone, which includes Santa Barbara, USA. For Bahia San Quintin and Estero de Punta Banda, MX, we assigned links for birds listed with hunting seasons and limits in the SEMARNAT working season document[6].

Fisheries. For the Carpinteria Salt Marsh, we referenced California Saltwater Sport and Angling fishing regulatory documents[3,7]. For the two salt marshes in Baja, Mexico, we referenced the National Fishing Charter provided by the Instituto Nacional de Pesca[4]. We assigned fishery links to any species that was reported as fished in the northern region of Baja, Mexico.

Table 1. Number of species providing each ecosystem service directly (i.e., redundancy).

Ecosystem Service	Number of species directly providing ecosystem service		
	Carpinteria Salt Marsh	Estero de Punta Banda	Bahai Falsa de San Quintin
Wave attenuation	6	9	10
Shoreline stabilization	8	10	13
Carbon sequestration	51	42	69
Water filtration	18	20	15
Fisheries	9	5	0
Bird watching	8	11	27
Waterfowl hunting	5	4	3

I.2 Species Loss Sequences

Ecosystem Service Providers: The species removed in this extinction sequence are the species directly providing ecosystem services. These include species that were identified in section 2.2 that were linked to services directly in the link data. For example, birds that were linked to waterfowl hunting and fish that were linked to fishing because they were listed in a regulatory document were removed in this extinction sequence. We first ran this sequence three times using different orders of species loss before deciding to use the biomass-ordered sequence discussed in the main text. We first filtered the interaction data to only include the species that provide services directly. We further simplified the data so that species that provide multiple services were only listed once. We simulated the loss of direct ecosystem service providers in three ways: 1. By biomass, in ascending order, 2. By abundance, in ascending order, and 3. Randomly, simulated 100 times. We estimated missing abundance and biomass data using previously published formulas[8]. Following this, we had biomass data for all species and abundance data for most species. Both food web and ecosystem service robustness were higher under the abundance-ordered sequence (see Figure S4). We chose to include the biomass- and random-ordered sequence in our final results because we had more data for these sequences. This extinction sequence did not include all the species, so the simulations ended when the last service provider was removed.

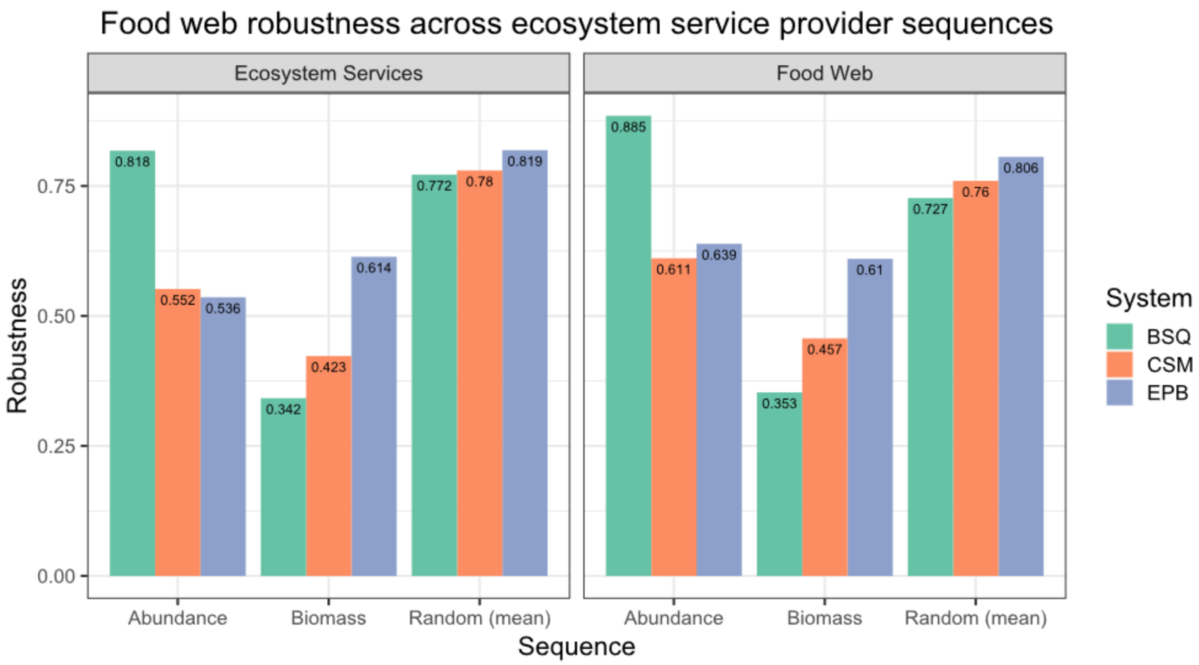

Figure 2. Robustness values vary for the removal of ecosystem service providers by biomass (low to high biomass) and abundance (least to most abundant) for A) the aggregate ES robustness (R_{ES}) and B) food web robustness (R_F). Results are shown for each system: BSQ, CSM, and EPM.

Supporting species: We calculated the personalized PageRank[9,10] score for each species in the food web, once for each ecosystem service (Figure S3 and S4). An important specification in a PageRank algorithm is α – the probability that the random walker terminates its walk at any given node (Figure S4). The magnitude of α determines how far the random walker gets into the network. The higher the value of α, the more likely the random walker terminates its walk closer to the starting node. Commonly, α is set to 0.15[9]. We tested various values of α to assess potential variation in the "importance" of supporting species, i.e. species with a high probability of the walker visiting that node. We calculated the personalized PageRank of all species for each ecosystem service using the following α values: 0.15, 0.35, and 0.45. We then calculated each species mean contribution to ecosystem services in aggregate (see main Methods for details) and calculated the robustness of the food webs and ecosystem services with the following sequences of species losses: high to low importance and low to high importance (for all three α values). We did not observe substantial variation in food web or ecosystem service robustness values (except see BSQ Food Web, α = 0.5; Figure S3), thus we followed commonly used α values and included the results from α = 0.15.

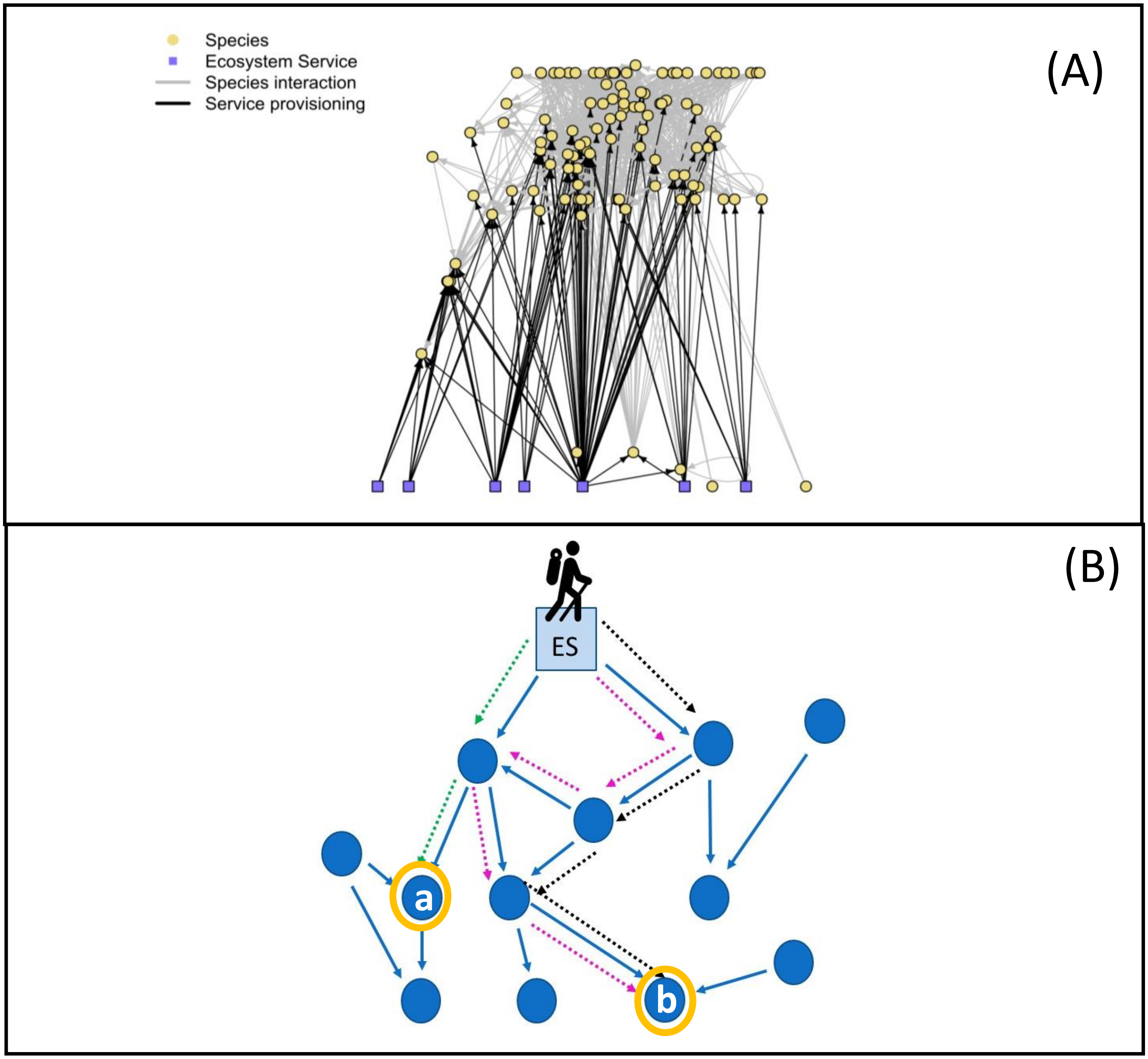

Figure 3. Conceptual example of applying the Personalized PageRank to identify supporting species. (A) Example of network data (Carpinteria Salt Marsh) with flipped edges for calculating Personalized PageRank to identify critical supporting species. Arrows point from ecosystem services to ecosystem service providers, and from consumers to resources. (B) Personalized PageRank allows us to specify the starting node as ecosystem service nodes. The walker starts every walk at the ecosystem service node, pursuing paths through the network at random. At each new node, the probability that the random walk terminates at a given node is α, and the probability that the walk continues is 1- α. Here, in this simplified diagram, 2/3 of the random walks end at node b, and 1/3 of the walks end at node a, indicating that node b would have a higher probability than a, and both have a higher probability than the rest of the nodes. The higher probability can be interpreted as “more important” to the ecosystem service node.

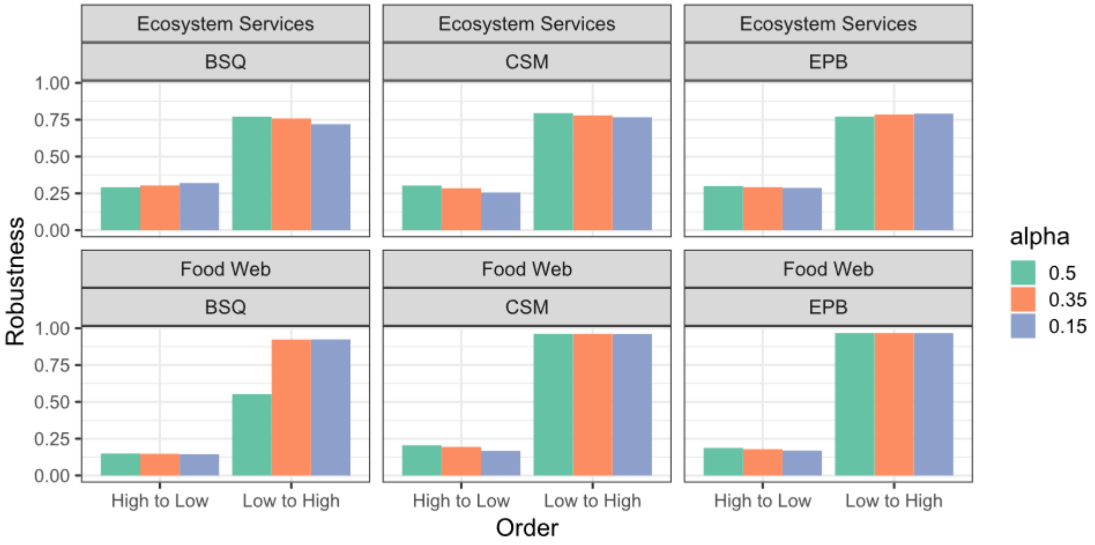

Figure 4. Robustness values varied little across the three levels of α used to identify supporting species. Top row shows ecosystem service (ES) robustness, considering all 7 ES in aggregate; bottom row shows food web robustness. The three columns are the three salt marshes (from left to right: Bahia Falsa de San Quintin (BSQ), Carpinteria Salt Marsh (CSM), Estero de Punta Banda (EPB). Alpha is the probability that the random walk terminates at any given node (α in Figure S3). Alpha (α) values ranged from 0.15-0.5, where values closer to 1 are high and values closer to 0 are low. The higher the value of alpha, the shorter the random walks (i.e., the random walker may not reach the basal trophic levels as frequently).

Rarity and body size: Previous food web robustness studies have simulated species loss ordered based on body size[11–13], which can influence extinction risk[14]. However, both large-bodied species and small-bodied species can be vulnerable to disturbances. We ran two body size sequences following the literature (removing from large to small and small to large bodied species[14,15]). We used body size information from the original food web data[1]. However, this sequence did not include plants because there is not plant body size information. To account for this, we instead included the Rarity sequence, where species are removed from low to high relative abundance (estimates based on Hechinger et al[1,8]). The order of the sequence was identical except for the difference in inclusion of plant species, so we decided to only include the rarity sequence in the main text.

Species impacted by system-specific threats, 'vulnerable species': Prior to conducting the literature synthesis to determine which species are threatened, we performed a literature search to identify salt marsh-specific threats. We searched Google Scholar using the following terms: salt marsh, estuary, disturbance(s), threat(s), risk(s). Once we established estuarine acidification, eutrophication and hypoxia, and pollution as our disturbance we searched for species in our food webs that are known to be impacted by them. We looked at each species individually using a combination of its scientific or common name and each of the disturbances. If a reference existed to support that species *s* displayed known mortality to the threat *i*, "1" was recorded to indicate the species was threatened, and the reference was recorded. We recorded "0" for all species that did not have a record of known mortality. While this method allowed us to systematically identify threatened species, we likely underestimated the number of threatened species. Some species are part of taxonomic groups that have varying impacts to these disturbances, such as fish. For these species, we only recorded "1" if the specific species name displayed known mortality. The species threatened by these stressors was a small subset of the full species list. Supplementary Data 1 includes the threatened species across the three estuaries and the reference supporting its categorization.

I.3 Robustness methods and sensitivity analyses

We simulated extinctions in R, relying on the package purr in tidyverse v 0.3.4[16]. The procedure is as follows: a target species is removed from the network, then any species who no longer has any resources in the network is considered to be secondarily extinct and are tallied. The process of target species removal continues until there are no remaining target species in the sequence.

In the main text, we calculate robustness as the area under the curve where the x-axis is the proportion of target species removed and the y-axis is proportion of species/ecosystem services that did not go secondarily extinct. We calculated the y-axis as:

$$\frac{S - C}{S}$$

where S is the number of nodes susceptible to secondary extinction (i.e., non-basal) and C is the cumulative number of secondary extinctions at each time step, t. Species nodes are susceptible if they have at least one resource (in-degree > 0, i.e., non-basal species), and all ecosystem services are susceptible because they inherently rely on at least one species (all in-degree > 0). This calculation allows us to only track secondary losses, regardless of whether the node represents a species or an ecosystem service. If the y-axis does not reach 0 in the food web, that indicates that some susceptible species were directly removed (i.e., primary extinctions on the x-axis), and/or that there are susceptible species remaining in the food web following the completion of a sequence.

We tested the sensitivity of our results to the choice of x-axis for calculating the Area Under the Curve (following [17]). Specifically, we examined multiple variations for the x-axis to make sure that the correlation between food web and ecosystem service robustness was consistent. The robustness values (AUC) varied based on the x-axis calculation, so it was important to test that the relationship between the two values was not sensitive to this calculation.

We calculated the robustness of the Carpinteria Salt Marsh (CSM) food web and ecosystem services for all 12 sequences using the following two equations to calculate the x-axis:

(1) $\frac{D}{L_{Seq}}$ where D is the number of species directly removed (i.e., number of realized primary extinctions), and L_{Seq} is the length of the intended sequence; and

(2) $\frac{D}{N}$ where D is the number of species directly removed (i.e., the number of realized primary extinctions), and N is the total number of species in the food web.

We then ran two Spearman's correlation tests on the food web and ecosystem service robustness for CSM, once using robustness values calculated with x-axis equation (1) above, and once using robustness values calculated from x-axis equation (2).

The main results remained unchanged: Food web and ecosystem service robustness are strongly and positively correlated for both equation (1) ($\rho[12]=0.93$, $p < 0.001$) and equation (2) ($\rho[12]=0.881$, $p < 0.001$). We used robustness values that used equation (1) for the x-axis because it better reflects the response of the food webs and ecosystem services to the specific order of species removals in each of the sequences.

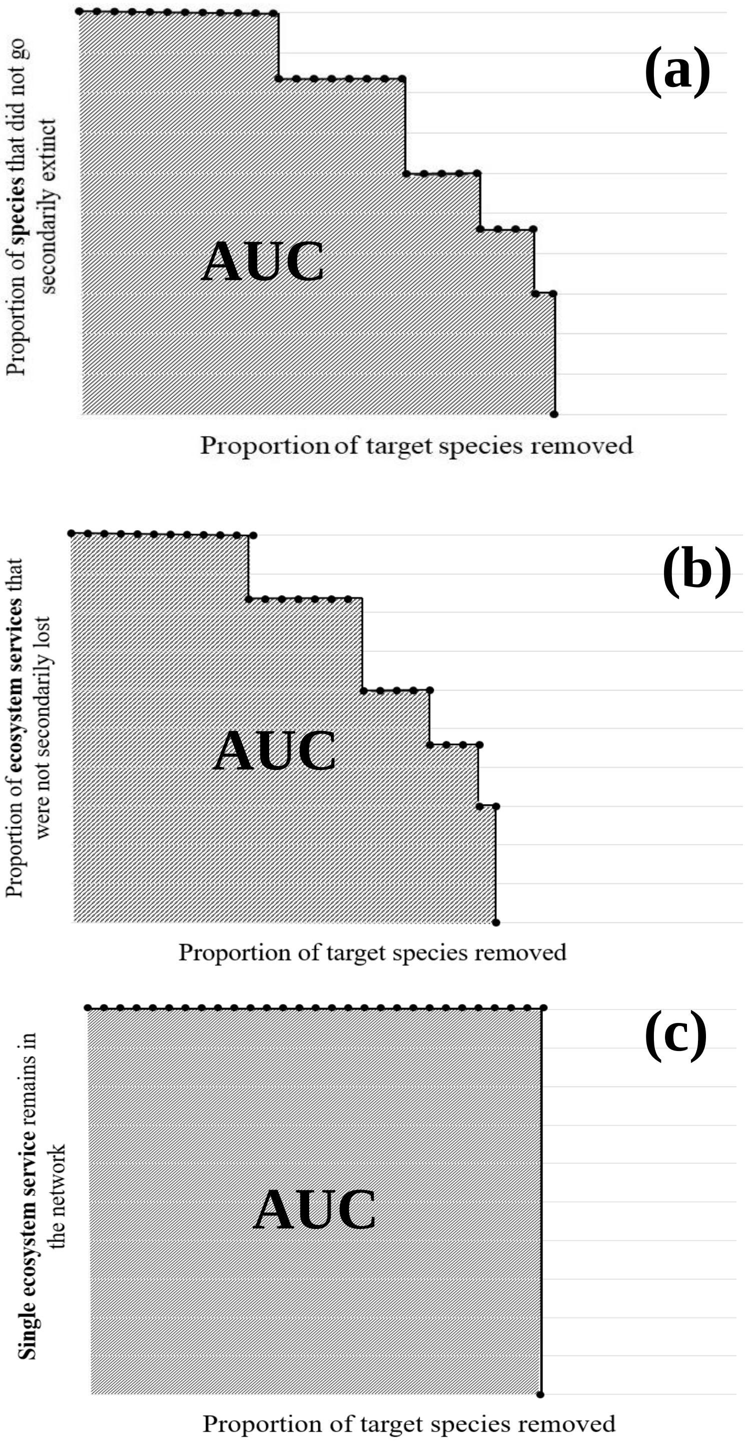

Figure 5. Illustration of unweighted robustness calculations for (a) food webs, (b) ecosystem services in aggregate, and (c) individual ecosystem services. In (a), (b), and (c), the *x*-axis is proportion of target species removed, which increases towards 1 every time a species is removed in a species loss sequence. In (a), the y-axis is proportion of *species* remaining following the removal of a species. In (b), the *y*-axis is the proportion of services (in aggregate, including all 7 services) remaining following the removal of a species. Each decrease in the y-axis shows when an ecosystem service was lost. In (c), the y-axis indicates whether a single ecosystem service remains (y=1) or was secondarily lost (y=0). The key difference is that in food web robustness, we are tracking secondary *species* losses that result from primary species removal, and in ecosystem service robustness, we are tracking *ecosystem service* losses (either in aggregate (b) or singularly (c)) that result from primary species removals.

I.4 Robustness of ecosystem services with weighted species contributions

Species contributions to many ecosystem services scale proportionally to their biomass (e.g., regulating and supporting services[18–20], but see [21]). For instance, species with higher biomass contribute more to carbon fixation and nutrient cycling than species with low biomass[22]. We extended the ecosystem service robustness calculations in the Robustness Analysis section (main text) to account for differences in species' contributions to each service, proportionate to an ecosystem service provider's biomass. First, we estimated biomass for species without data because the original data[1] do not include biomass estimates for all species. To do this, we used methods explained in [8] where biomass is a function of species' abundances and body sizes:

$$biomass = abundance \times body\ size(kg)$$

Then, we simulated the same 12 extinction scenarios but tracked the *proportional* change in ecosystem services (Figure S6(a)) as opposed to the *binary* outcomes of retention or loss (i.e., complete service loss when all ecosystem service providers are lost). We calculated proportional changes in ecosystem service provision as an aggregate (Figure S6(a)) and as individual services (Figure S6(b)). The size of all ecosystem services was considered to be equivalent to the summed biomasses of all the species directly providing them. The key difference between the aggregate and individual weighted robustness is that the aggregate pools all ecosystem service providers' biomasses together, regardless of the service; and the individual calculation only considers species that are directly linked to a single service. For the aggregate, the proportion of ecosystem services remaining at each time step was calculated using:

$$\frac{ecosystem\ service\ provider\ biomass\ remaining}{total\ ecosystem\ service\ provider\ biomass}$$

With this calculation, each time an ecosystem service provider is lost, the y-axis decreases proportionally to its biomass. We calculated the weighted, individual ecosystem service robustness in the same way, except the y-axis only considers the ecosystem service providers directly linked to that service (Figure S6(b)). So, the denominator is the total ecosystem service provider biomass for the single ecosystem service, and the numerator is the remaining ecosystem service biomass for that single service (i.e., 1-cumulative sum of ecosystem service provider biomass removed or secondarily lost). Each time an ecosystem service provider is lost, the y-axis decreases proportionally to its biomass.

We did not do weighted contributions of species for birdwatching because the value of this service does not scale proportionally with a species' biomass[19,21,23].

Figure 6 Illustration of weighted robustness calculations for (a) ecosystem services in aggregate and (b) individual ecosystem services. In (a) and (b), the *x*-axis is the proportion of target species removed, which increases towards 1 every time a species is removed in a species loss sequence (as in the unweighted robustness calculation in X). In (a), the *y*-axis is the proportion of all services in aggregate (including all services except birdwatching) remaining following the removal and/or the secondary loss of an ecosystem service provider. Each decrease in the *y*-axis shows the percent of all ecosystem services that were lost, which is based on the biomass of the ecosystem service providers that were removed or secondarily lost. In (b), the *y*-axis indicates decreases in a single ecosystem service, which is based on the proportion of all ecosystem service providers biomass for that single ecosystem service. Each time an ecosystem service provider is lost for that ecosystem service, the *y*-axis decreases proportionally with that species' biomass.

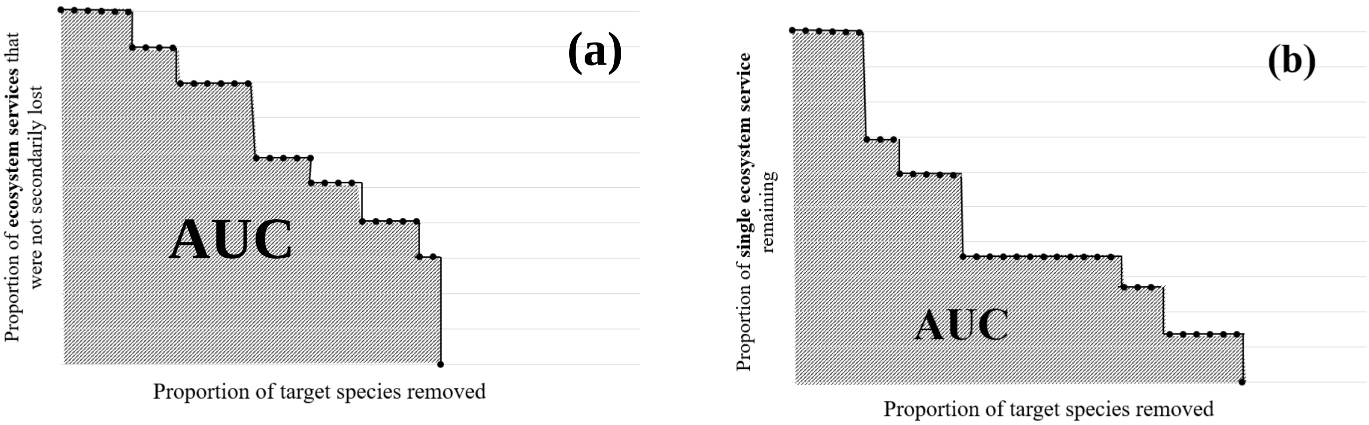

II. Supplementary Discussion

Figure 7 Food web (I) and ecosystem service (II) responses to primary species removal result in different rates of secondary losses. Each box shows the sequential loss of species and/or ecosystem services for 3 of the 12 species loss sequences: (A) Most to least connected, (B) Rarity, and (C) Vulnerable species. The three colors show the three salt marsh systems: Bahia Falsa de San Quintin (gold), Carpinteria Salt Marsh (blue) and Estero de Punta Banda (green). L_{seq} indicates the length of each sequence (the denominator in the x-axis). Note that while there were very few, if any, secondary losses in the Vulnerable species sequence, that only 5-20 species were removed, so the robustness values only reflect the robustness of the food web and the ecosystem services to the loss of those specific species.

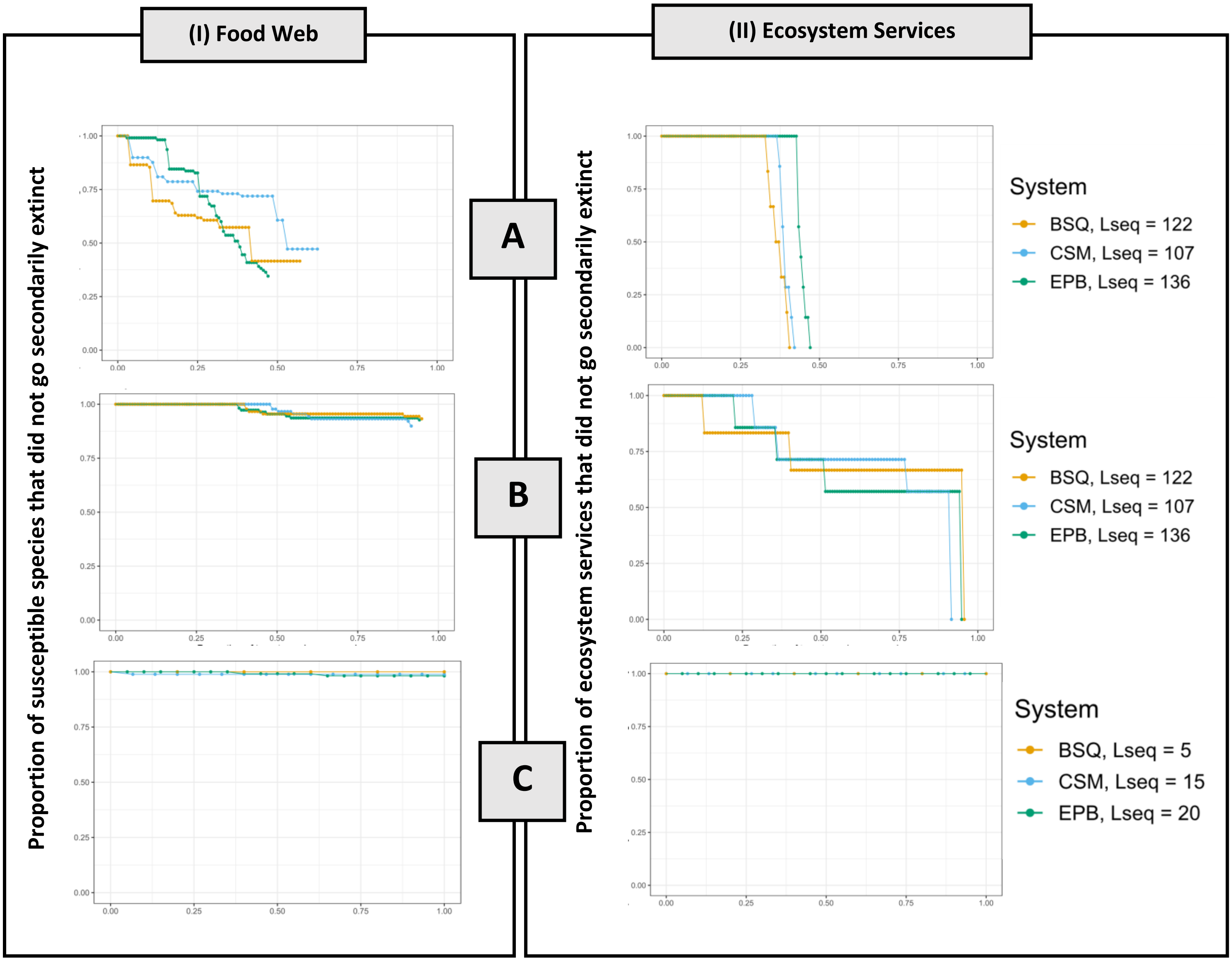

Figure 8. Aggregate ecosystem service robustness is generally lower when considering species' unequal contributions to ecosystem services (proportional to a species' biomass) across the 12 sequences: (A) Direct ecosystem service providers (high to low biomass), (B) Direct ecosystem service providers (low to high biomass), (C) Direct ecosystem service providers (random), (D) Most to least connected, (E) Random (maximum), (F) Random (minimum), (G) Random (mean), (H) Rarity, (I) Supporting species (most to least important), (J) Supporting species (least to most important), (K) Supporting species (random), (L) Vulnerable species. Weighted and unweighted ecosystem service robustness are highly positively correlated ($r_s[36] = 0.760$, $p < 0.001$).

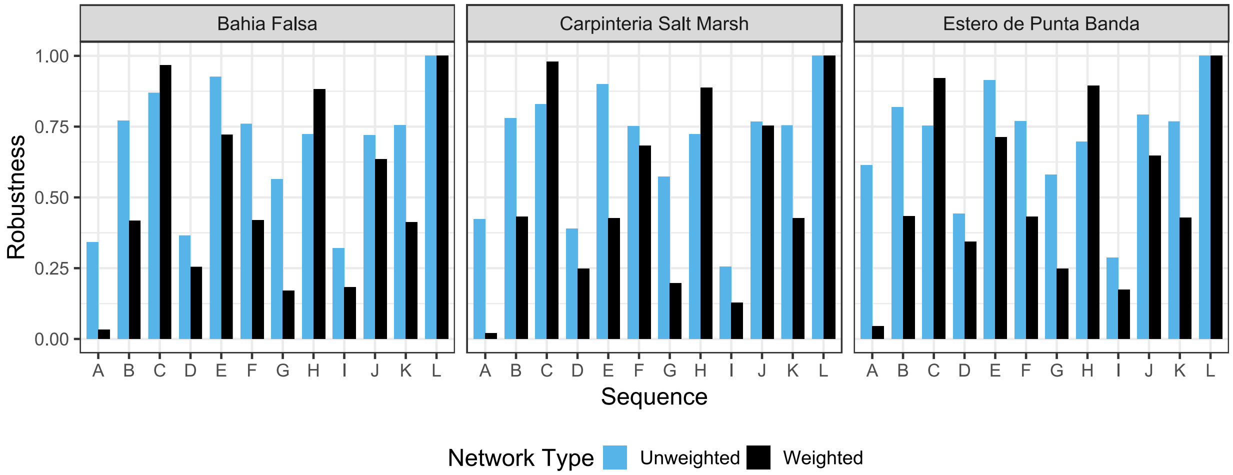

Figure 9. Food web and weighted ecosystem service robustness remain strongly and positively correlated ($r_s[36] = 0.885$, $p < 0.001$)**.** A comparison of food web robustness (unweighted) vs. weighted ecosystem service robustness across each species loss scenario and salt marsh system. The black line is a regression line. The shape of data points represents the salt marsh system: Bahia Falsa de Sant Quintin (Figure 1C), Carpinteria Salt Marsh (Figure 1A), and Estero de Punta Banda (Figure 1B). The color denotes the sequence of species removals: ecosystem service providers (removed by biomass and randomly), supporting species (removed by high-to-low importance, low-to-high importance, and randomly), most-to-least connected, randomly (reporting: highest, lowest and mean), rarity (removed by relative abundance - from least to most abundant), and vulnerable species (removed from least to most abundant).

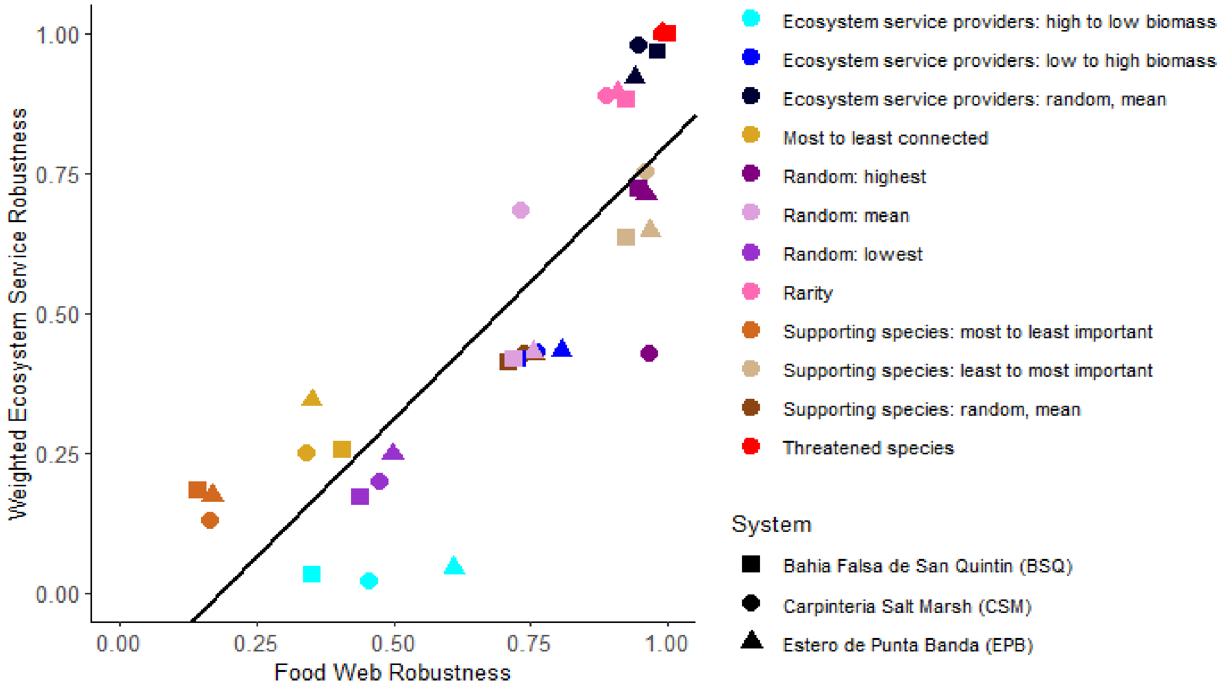

II.1 Food web and ecosystem service robustness strong, positive correlation not skewed by the length of the vulnerable species sequence.

We ran sequences (i.e., species loss scenarios) that varied in length, where some ended after very few species were removed and others ended when all species were removed. The vulnerable species sequence was short (L_{seq} ranged from 5-20 across the 3 salt marsh systems), due to our conservative approach to include species in the sequence (see main text). This short sequence length could mirror reality because it is not realistic that all species are always lost. The removal of vulnerable species (based on our methods for assigning threat status) did not cause many (if any) secondary extinctions, resulting in high food web and ecosystem service robustness. However, we wanted to be sure that these high (and very similar) robustness values did not skew our correlation tests (e.g., artificial strong correlation). To test this, we ran two correlation tests on the food web and ecosystem service robustness values from Carpinteria Salt Marsh (CSM): (1) data from all sequences, including the vulnerable species data, and (2) data from all sequences, excluding the vulnerable species data. The main results remain unchanged. The correlation between food web and ecosystem service robustness was strong, positive and significant (with $p < 0.001$) when the vulnerable species sequence was included ($\rho[36]=0.884$) and excluded ($\rho[33]=0.849$), so we included it in our main results.

II.2 Ecosystem service robustness across sequences and systems

Ecosystem service robustness was varied across all sequences (min. R_{ES} = 0.256, max. R_{ES} = 1; main Figure 4(a)). Ecosystem service robustness was highest (R_{ES} = 1) for all three systems under the system-specific threats sequence because no services were lost after all species were removed. The system-specific threats sequence was the shortest sequence (with 5-20 species directly removed). Ecosystem service robustness was lowest for all three systems under the most-to-least important supporting species sequence (min. R_{ES}=0.256, max. R_{ES}=0.320). Ecosystem service robustness varied across systems (difference in min. and max. R_{ES} ranged from 0-0.261).

II.3 Food web robustness across sequences

Food web robustness varied across all 12 sequences of species loss (min. R_F = 0.145, max. R_F = 1; main Figure 4(b)). Under the most-to-least connected and the most-to-least important supporting species sequences, the food web collapsed before more than 50% of the species were removed directly (main Figure 5C) Similarly, the random removal of species resulted in secondary species losses sooner than the threat-based sequences and the important ecosystem service provider sequence.

II.4 Sensitivity of results to sequences that removed different numbers of species

We simulated sequences of varying lengths, ranging from 5 species to all species. Table S2 shows whether or not a sequence included the full length of the species list. We only compared the raw robustness value for sequences that included the full species list.

Table 2. **Length of species loss sequences.** Some sequences included the entire species list at each salt marsh system, while others only included a subset of the species.

Sequence	Full species list or subset?
Ecosystem service providers	Subset of species list
Supporting species	Full species list
Rare species	Full species list
Vulnerable species	Subset of species list
Most to least connected	Full species list
Random	Full species list

To compare the short sequence, ‘Vulnerable species’, to the other sequences of species lost, we compared the raw number of species that were secondarily lost in the removal of the same number of target species for the other sequences. We found that there were varying levels of secondary species losses across the sequences in the same number of target removals (Table S3).

Table 3. Number of secondary species losses per sequence in the same number of target removals as the Vulnerable species sequence. Each salt marsh has a different number *n* of species in the vulnerable species sequence: 15 in Carpinteria Salt Marsh (CSM), 5 in Bahia Falsa de San Quintin (BSQ), and 20 in Estero de Punta Banda (EPB). We looked at the number of species that were secondarily lost in each sequence for the first *n* species removals.

System	Number removed in vulnerable species sequence	Sequence	Number secondary losses
CSM	15	Vulnerable species	0
CSM	15	Most to least connected	5
CSM	15	Rarity	0
CSM	15	Eco. Serv. Provider (high to low biomass)	19
CSM	15	Eco. Serv. Provider (low to high biomass)	0
CSM	15	Supporting Spp. (most to least important)	31
CSM	15	Supporting Spp. (least to most important)	0
BSQ	5	Vulnerable species	0
BSQ	5	Most to least connected	0
BSQ	5	Rarity	0
BSQ	5	Eco. Serv. Provider (high to low biomass)	12
BSQ	5	Eco. Serv. Provider (low to high biomass)	0
BSQ	5	Supporting Spp. (most to least important)	28
BSQ	5	Supporting Spp. (least to most important)	0
EPB	20	Vulnerable species	2
EPB	20	Most to least connected	2
EPB	20	Rarity	0
EPB	20	Ecosystem Serv. Provider (high to low biomass)	24
EPB	20	Ecosystem Serv. Provider (low to high biomass)	0
EPB	20	Supporting Spp. (most to least important)	42
EPB	20	Supporting Spp. (least to most important)	0

II.5 Is ecosystem service robustness a function of sample size?

Given that ecosystem service robustness differed from food web robustness, and that this approach has not yet been applied, we explored the extent to which our ecosystem service robustness results was a function of sample size (three salt marsh systems, 7 ecosystem services each). By matching each ecosystem service to a similar species (in-degree centrality and trophic level), we are able to explore how are results compare to a common unit in robustness studies – species.

We calculated the in-degree centrality and trophic level of all nodes in the CSM food web-ES network using iGraph v 1.2.5[10] and NetIndicies v 1.4.4[24] R packages, respectively. Once we had this information, we identified 2 species nodes that closely matched the in-degree centrality and/or the trophic level of each of the 7 ecosystem services (Table S4). We then ran the same 14 robustness sequences (but with these 7 matching species removed from the sequences) and calculated the overall robustness of the 7 identified species (in aggregate, Table S5). Finally, we calculated the mean number of species remaining when each ecosystem service was lost, and the mean number of species that were lost prior to losing each ecosystem service. We did this for the 7 matched species as well (Table S6).

From this sensitivity analysis, we see that species similar to ecosystem services (in terms of in-degree centrality and trophic level) behave similarly to ecosystem services themselves when species are removed in different orders. However, the robustness of species similar to ecosystem services is higher or lower than ecosystem service robustness depending on the sequence of species removals. Further, ecosystem service nodes and the species similar to ecosystem services were lost at different times in each sequence (Table S6, Figure S8). They similarly display threshold behavior (Figure S8).

Table 4. Selecting species that match ES in trophic level and in-degree centrality We calculated the in-degree centrality and trophic level for each ecosystem service and species in CSM. Then, we selected 7 species that had both similar trophic level and in-degree centrality to each ES that we later tracked to calculate the robustness.

Ecosystem Service Node (ES)	In-degree Centrality (ES)	Trophic Level (ES)	Species Node (SPP)	In-degree Centrality (SPP)	Tropic Level (SPP)
Wave Attenuation (350)	6	2	213	5	2
Shoreline Stabilization (450)	8	2	116	9	3.66
Carbon Sequestration (550)	51	3.21	86	43	4.49
Water Filtration (650)	18	2.81	119	18	3.15
Fishing (750)	9	3.98	173	10	3.19
Bird watching (850)	8	4.87	210	8	3.89
Waterfowl hunting (950)	5	3.96	208	5	3.64

Table 5: Comparing the robustness of ES and species with similar trophic level and centrality. We calculated the robustness of the ecosystem service nodes and the species with similar metrics for each of the 12 extinction sequences.

Sequence of species lost	ES robustness	Species with similar metrics robustness
Most to least connected	0.389	0.425
Random (mean)	0.7518919	0.7659114
Random (min)	0.5734312	0.5164286
Random (max)	0.8991989	0.9264286
Rarity	0.722964	0.6922563
Vulnerable species	1	1
Direct ESP – low to high biomass	0.829241	0.8973214
Direct ESP – high to low biomass	0.4229911	0.4508929
Direct ESP – Random (mean)	0.7800446	0.7547768
Species Supporting ES – most to least important	0.2556742	0.2503338
Species Supporting ES – least to most important	0.7670227	0.8898531
Species Supporting ES – Random (mean)	0.7549933	0.7138985

Table 6. Tracking when ecosystem service nodes and matching species nodes are lost. For each ecosystem service node and matching species nodes (target nodes), we tracked how many species were lost and remaining when each target node was secondarily lost. Using these raw numbers for each of the 12 sequences, we calculated the mean proportion of species lost and remaining, where the denominator is the total number of species in the system. This allowed us to compare the sensitivity of the ecosystem service nodes and the matching species to each sequence of species loss.

Ecosystem Service (ES)	Mean prop. spp remaining when lost (sd)	Mean prop. spp lost when ES lost (sd)	Species with similar metric (SPP)	Mean prop. spp remaining when lost (sd)	Mean prop. spp lost when lost (sd)
**Wave Attenuation (350)**	0.19 (0.24)	0.87 (0.24)	**213**	0.33 (0.27)	0.73 (0.27)
**Shoreline Stabilization (450)**	0.16 (0.25)	0.90 (0.24)	**116**	0.19 (0.15)	0.87 (0.15)
**Carbon Sequestration (550)**	0.05 (0.09)	0.95 (0.09)	**86**	0 (0)	1 (0)
**Water Filtration (650)**	0.14 (0.23)	0.93 (0.22)	**119**	0.21 (0.16)	0.86 (0.16)
**Fishing (750)**	0.21 (0.09)	0.85 (0.09)	**173**	0 (0)	1 (0)
**Bird watching (850)**	0.34 (0.31)	0.72 (0.31)	**210**	0.20 (0.16)	0.86 (0.16)
**Waterfowl hunting (950)**	0.43 (0.31)	0.64 (0.21)	**208**	0.32 (0.24)	0.74 (0.24)

Figure 10. **Species losses causing the secondary loss of ecosystem services and species similar to ecosystem services.** We calculated the robustness of ecosystem service nodes and species nodes with similar trophic level and in-degree centrality using the data from these plots. Each box shows the sequential loss of species (*x*-axis) and the secondary losses of ecosystem services (*y*-axis, blue) or species similar to ecosystem services (*y*-axis, yellow).

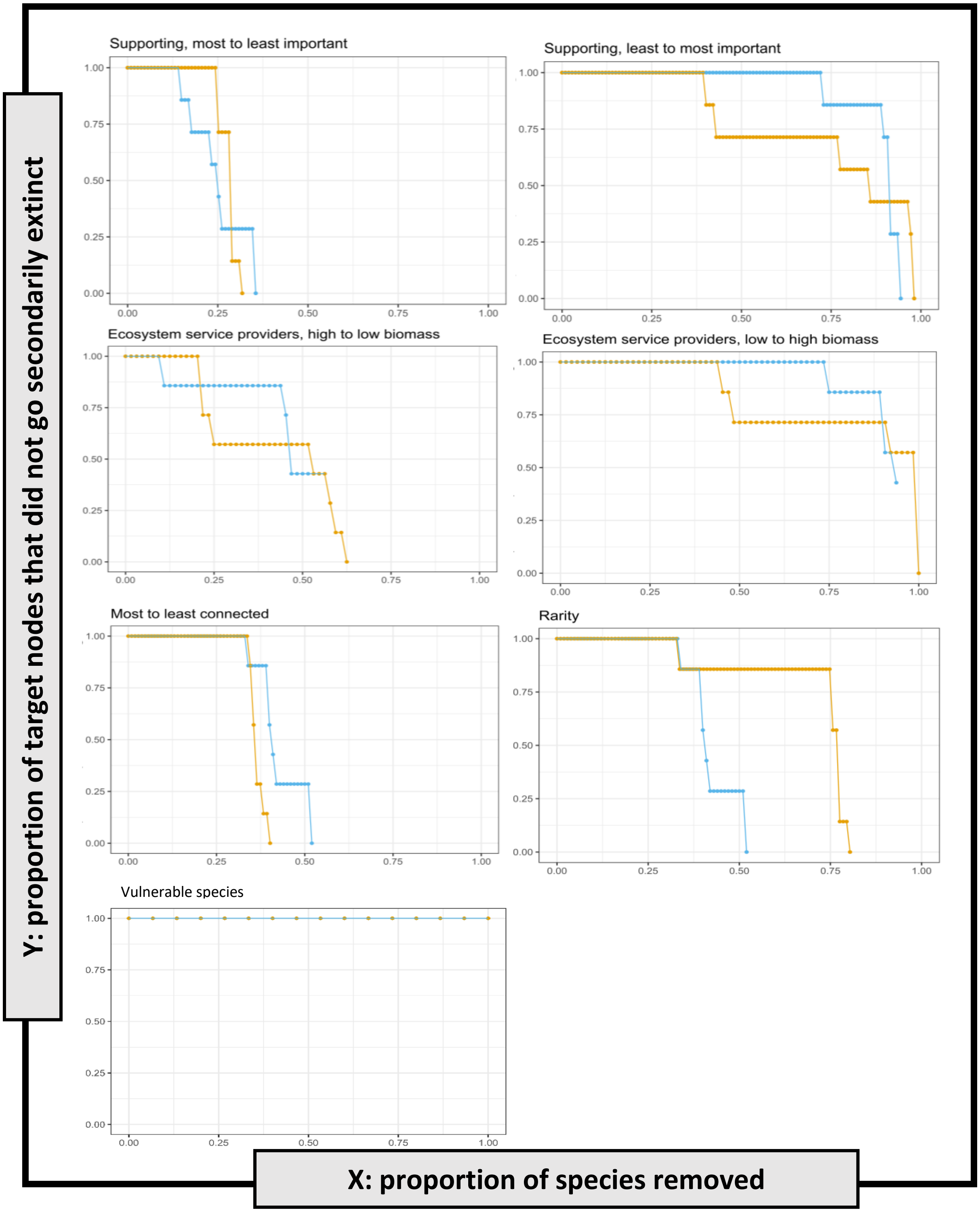

II.6 Sensitivity of results to the exclusion of parasites

Parasites play an important and unique role in estuarine foods webs[25,26], altering their structure[27] and stability[28]. Previous studies in the three estuaries under consideration here[1] have demonstrated that the inclusion of parasites can decrease network robustness[29]. In these estuaries, the susceptibility of parasites to secondary extinctions is a result of their complex life histories which require them to pass through a series of hosts in specific sequence in order to complete their life cycle. Parasites in these estuaries are dominated by trematodes, many of which must pass through a single species of snail as larva, but which can occur in dozens of species of fish-eating birds as adults. Thus, the parasite influences on robustness are a result of ontogenetic diet shifts (often serial specialization) which can be captured by stage-structured analysis. While parasites have stage-specific diet information in our networks, their free-living counterparts (which comprise the majority of species) do not. This precluded stage-structured analyses. Given their potential robustness impacts, we incorporated adult parasites and re-ran our robustness analyses for the food webs and ecosystem services for all sequences on networks that included parasites and parasitic interactions (see Table S8). To examine the sensitivity our results to including or excluding parasitic interactions, we ran a Spearman's correlation test on the food web and ecosystem service for all 3 systems and all 12 sequences.

Table 7. All three food webs increased in size when parasites were included. The number of species and number of links increased. Species-species links include non-trophic parasitic interactions. Total number of links includes species-species links and species-service links.

System	Number of species	Number of species-species links	Total number of links (including services)
Bahia Falsa	161	3759	3896
Carpinteria	151	3660	3765
Estero de Punta Banda	198	5531	5632

Including parasites in the networks, food web robustness remained strongly, and positively correlated with ecosystem service robustness ($r_s[36]=0.738$, $p=2.8e\text{-}07$; Figure S11). Generally (for more than 6 of the 12 sequences), robustness values were higher when parasites were included for both the food webs and the ecosystem services (across all three systems; Figure S12). This is due to the fact that we only included adult life stages, which for these parasites tend towards diet generality inflating robustness[25,29]. Interestingly, food web robustness was substantially higher when parasites were included in all three salt marshes when supporting species were removed from most to least important (Figure S12: 'I' Food Web). However, similar to previous studies that have compared food web robustness with and without parasites on these food webs, robustness decreased for both food webs and ecosystem services when parasites were included for two sequences: most to least connected[26] and random[28,29].

Figure 11. Food web and ecosystem service robustness remain highly and positively correlated when parasites are included ($r_s[36] = 0.738$, $p < 0.001$). A comparison of food web vs. ecosystem service robustness across each species loss scenario and salt marsh system. The black line shows the linear regression line. The shape of data points represents the salt marsh system: Bahia Falsa de San Quintin, Carpinteria Salt Marsh, and Estero de Punta Banda. The color denotes the sequence of species removals: ecosystem service providers (removed by biomass and randomly), supporting species (removed by high-to-low importance, low-to-high importance, and randomly), most-to-least connected, randomly (reporting: highest, lowest and mean), rarity (removed by relative abundance - from least to most abundant), and vulnerable species (removed from least to most abundant).

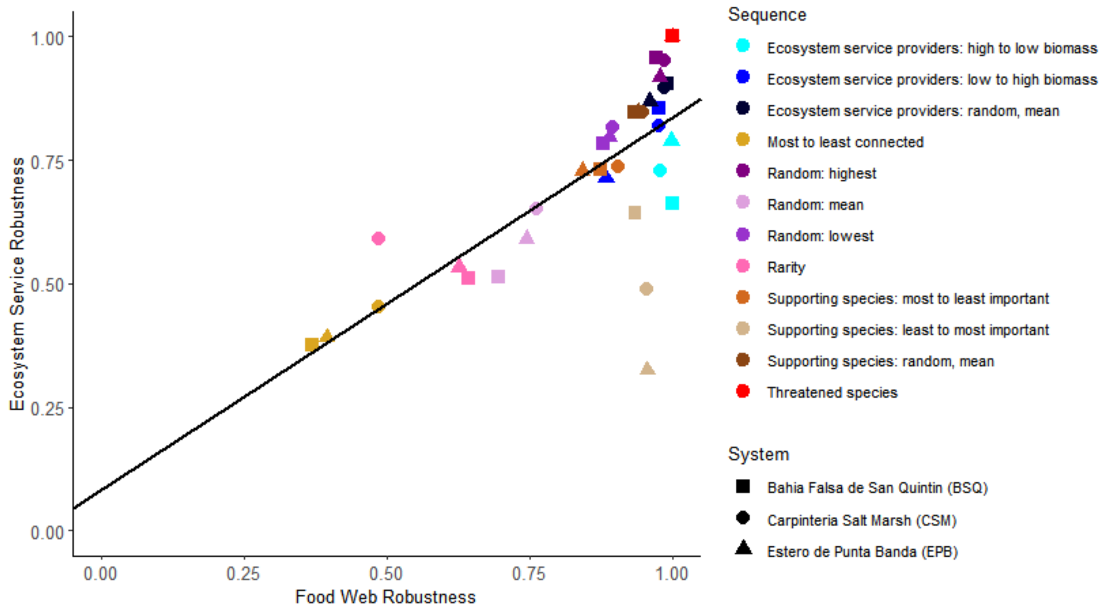

Figure 12. Robustness values generally increase when parasites are included versus excluded for both the food webs and the ecosystem services across the 12 sequences: (A) Direct ecosystem service providers (high to low biomass), (B) Direct ecosystem service providers (low to high biomass), (C) Direct ecosystem service providers (random), (D) Most to least connected, (E) Random (maximum), (F) Random (minimum), (G) Random (mean), (H) Rarity, (I) Supporting species (most to least important), (J) Supporting species (least to most important), (K) Supporting species (random), (L) Vulnerable species.

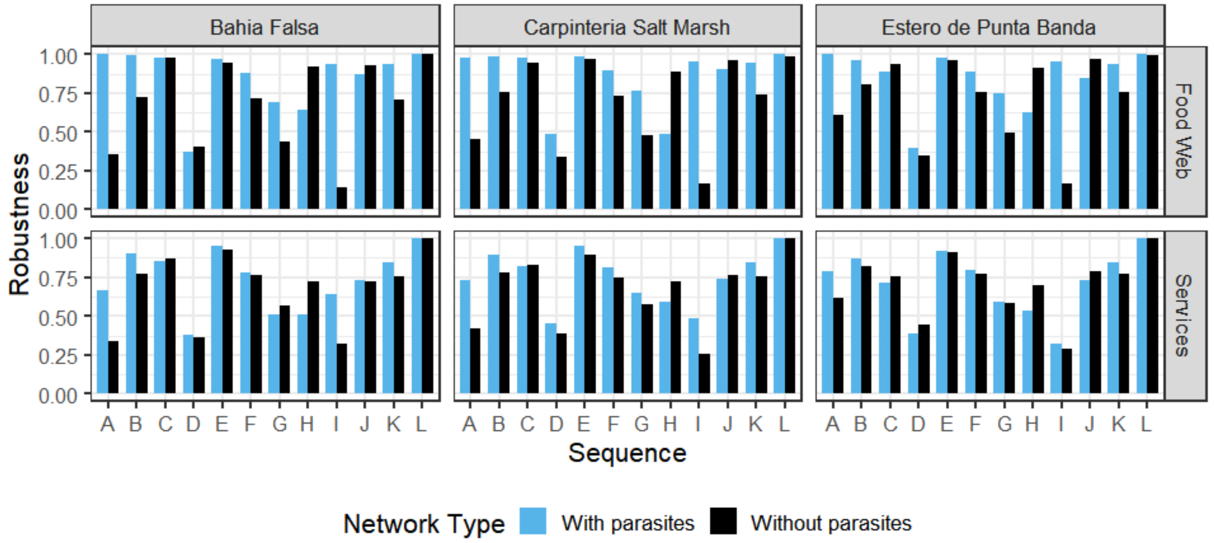

II.7 Individual ecosystem service robustness is driven by trophic level and redundancy

Table 8. Coefficient estimates and model summary for each of the 7 linear models. We fit 1 model of each sequence B-D and 1 model that included the data for all sequences. We did not make adjustments for multiple comparisons. The dependent variable in each model was individual ecosystem service robustness (R_{indiv}) and the independent variables were mean trophic level and link redundancy. Model: A) All sequences, B) Most-to-least connected, C) Ecosystem service providers (high-to-low biomass), D) Ecosystem service providers (low-to-high biomass), E) Supporting species (most-to-least important), F) Supporting species (least-to-most important), G) Rarity.

**Model**	**A**	**B**	**C**	**D**	**E**	**F**	**G**
	**All sequences**	**Most-to-least connected**	**Ecosystem service providers (high-to-low biomass)**	**Ecosystem service providers (low-to-high biomass)**	**Supporting species (most-to-least important)**	**Supporting species (least-to-most important)**	**Rarity**
Intercept	0.715 *** (0.066)	0.365 *** (0.021)	0.029 (0.085)	1.163 *** (0.076)	0.283 *** (0.018)	1.051 *** (0.104)	1.116 *** (0.090)
Trophic Level	-0.060 * (0.025)	0.014 (0.008)	0.171 *** (0.032)	- 0.192 *** (0.029)	- 0.006 (0.007)	- 0.172 *** (0.039)	- 0.234 *** (0.034)
Link Redundancy	0.003 (0.001)	0.0004 (0.0005)	0.004 * (0.002)	0.004 * (0.002)	0.001 * (0.0004)	0.005 (0.002)	0.006 *** (0.002)
R^2	0.067	0.165	0.646	0.754	0.286	0.587	0.773
Adj. R^2	0.053	0.067	0.604	0.725	0.202	0.538	0.746
Num. Obs.	140	20	20	20	20	20	20

*** p < 0.001; ** p < 0.01; * p < 0.05

III. Supplementary References

1. Hechinger, R. F. *et al.* Food webs including parasites, biomass, body sizes, and life stages for three California/Baja California estuaries. *Ecology* **92**, 791 (2011).

2. Xiao, H. *et al.* Win-wins for biodiversity and ecosystem service conservation depend on the trophic levels of the species providing services. *J. Appl. Ecol.* **55**, 2160–2170 (2018).

3. CDFW. Sport fishing regulations. 12–14 (2018).

4. Diario Oficial de la Federación. Carta Nacional Pesquera. *D. Of. la Fed.* (2017).

5. Strimas-Mackey, M., Miller, E., Hochachka, W. & Cornell Lab of Ornithology. *eBird Data Extraction and Processing in R [R package auk version 0.3.2]*. (Comprehensive R Archive Network (CRAN), 2019).

6. Concesión, S. D. E. Subsecretaria de gestion para la protección ambiental. (2018).

7. CDFW. Angling Regulations Excerpts for 2018-2019. 2801 (2019).

8. Hechinger, R. F., Lafferty, K. D., Dobson, A. P., Brown, J. H. & Kuris, A. M. A common scaling rule for abundance, energetics, and production of parasitic and free-living species. *Science (80-.).* (2011). doi:10.1126/science.1204337

9. Bryan, K. & Leise, T. The Linear Algebra behind Google. *SIAM Rev* **3**, 13 (2009).

10. Csardi, G. & Nepusz, T. The igraph software package for complex network research. (2006).

11. Curtsdotter, A. *et al.* Robustness to secondary extinctions: Comparing trait-based sequential deletions in static and dynamic food webs. *Basic Appl. Ecol.* **12**, 571–580 (2011).

12. De Visser, S. N., Freymann, B. P. & Olff, H. The Serengeti food web: Empirical quantification and analysis of topological changes under increasing human impact. *J. Anim. Ecol.* **80**, 484–494 (2011).

13. Jacob, U. *et al. The Role of Body Size in Complex Food Webs. A Cold Case*. *Advances in Ecological Research* **45**, (2011).

14. Sodhi, N. S., Brook, B. W. & Bradshaw, C. J. A. Causes and Consequences of Species Extinctions. in *The Princeton Guide to Ecology* (2014). doi:10.1515/9781400833023.514

15. Ripple, W. J. *et al.* Extinction risk is most acute for the world’s largest and smallest vertebrates. *Proc. Natl. Acad. Sci. U. S. A.* (2017). doi:10.1073/pnas.1702078114

16. Henry, L. & Wickham, H. Purrr: Functional programming tools. *R Packag. version 0.3.2* (2019).

17. Bane, M. S., Pocock, M. J. O. & James, R. Effects of model choice, network structure, and interaction strengths on knockout extinction models of ecological robustness. *Ecol. Evol.* **8**, 10794–10804 (2018).

18. Balvanera, P., Kremen, C. & Martínez-Ramos, M. Applying community structure analysis to ecosystem function: Examples from pollination and carbon storage. *Ecol. Appl.* (2005). doi:10.1890/03-5192

19. Gaston, K. J. *et al.* Population Abundance and Ecosystem Service Provision: The Case of Birds. *Bioscience* **68**, 264–272 (2018).

20. Pau, S. & Dee, L. E. Remote sensing of species dominance and the value for quantifying

ecosystem services. *Remote Sens. Ecol. Conserv.* (2016). doi:10.1002/rse2.23

21. Dee, L. E. *et al.* When Do Ecosystem Services Depend on Rare Species? *Trends Ecol. Evol.* **xx**, 1–13 (2019).

22. Davies, T. W. *et al.* Dominance, biomass and extinction resistance determine the consequences of biodiversity loss for multiple coastal ecosystem processes. *PLoS One* (2011). doi:10.1371/journal.pone.0028362

23. Booth, J. E., Gaston, K. J., Evans, K. L. & Armsworth, P. R. The value of species rarity in biodiversity recreation: A birdwatching example. *Biol. Conserv.* **144**, 2728–2732 (2011).

24. Kones, J. K., Soetaert, K., van Oevelen, D. & Owino, J. O. Are network indices robust indicators of food web functioning? A Monte Carlo approach. *Ecol. Modell.* (2009). doi:10.1016/j.ecolmodel.2008.10.012

25. Lafferty, K. D. *et al.* Parasites in food webs: the ultimate missing links. *Ecol. Lett.* **11**, 533–46 (2008).

26. Lafferty, K. D. & Kuris, A. M. Parasitic castration: the evolution and ecology of body snatchers. *Trends in Parasitology* (2009). doi:10.1016/j.pt.2009.09.003

27. Dunne, J. A. *et al.* Parasites Affect Food Web Structure Primarily through Increased Diversity and Complexity. *PLoS Biol.* **11**, 1–17 (2013).

28. Lafferty, K. D. & Kuris, A. M. Parasites reduce food web robustness because they are sensitive to secondary extinction as illustrated by an invasive estuarine snail. *Philos. Trans. R. Soc. B Biol. Sci.* **364**, 1659–1663 (2009).

29. Rudolf, V. H. W. & Lafferty, K. D. Stage structure alters how complexity affects stability of ecological networks. *Ecol. Lett.* **14**, 75–79 (2011).
