# Supplementary material for: An ecological network approach to predict ecosystem service vulnerability to species losses
Source: Nat Commun. 2021 Mar 11;12:1586. doi: 10.1038/s41467-021-21824-x (PMC7952599; doi:10.1038/s41467-021-21824-x)
Supplement: Supplementary file 3 — Description of Additional Supplementary Files [file 41467_2021_21824_MOESM3_ESM.pdf]

Description of Additional Supplementary Files

File Name: Supplementary Data 1

Description: Filtered Species Node list for the three salt marsh systems: BSQBahia Falsa de San Quintin, CSM-Carpinteria Salt Marsh, EPB-Estero de Punta Banda. SpeciesID indicates the final species that were included in our analysis. These SpeciesIDs can be merged with the previously published salt marsh food web data (see Hechinger et al. 2011, Ecology Archives E092-066) to obtain species trait data.

File Name: Supplementary Data 2

Description: Filtered Edgelists for species trophic interactions in the three salt marsh systems (System): BSQ-Bahia Falsa de San Quintin, CSM-Carpinteria Salt Marsh, EPB-Estero de Punta Banda. ResourceSpeciesID indicates the prey in the trophic interaction. The "from" in the directed edge. ConsumerSpeciesID indicates the predator or consumer in the trophic interaction. The "to" in the directed edge. Type indicates that it is a Feeding (trophic) interaction. This edge list only includes species interactions. o

File Name: Supplementary Data 3

Description: Final list of vulnerable species by system.
