# Supplementary material for: An ecological network approach to predict ecosystem service vulnerability to species losses
Source: Nat Commun. 2021 Mar 11;12:1586. doi: 10.1038/s41467-021-21824-x (PMC7952599; doi:10.1038/s41467-021-21824-x)
Supplement: Supplementary file 7 — Reporting Summary [file 41467_2021_21824_MOESM7_ESM.pdf]

nature research

Corresponding author(s): Aislyn Keyes

Last updated by author(s): Jan 13, 2021

Reporting Summary

Nature Research wishes to improve the reproducibility of the work that we publish. This form provides structure for consistency and transparency in reporting. For further information on Nature Research policies, see our Editorial Policies and the Editorial Policy Checklist.

Statistics

For all statistical analyses, confirm that the following items are present in the figure legend, table legend, main text, or Methods section.

n/a	Confirmed	
☐	☒	The exact sample size (*n*) for each experimental group/condition, given as a discrete number and unit of measurement
☒	☐	A statement on whether measurements were taken from distinct samples or whether the same sample was measured repeatedly
☐	☒	The statistical test(s) used AND whether they are one- or two-sided<br>*Only common tests should be described solely by name; describe more complex techniques in the Methods section.*
☐	☒	A description of all covariates tested
☐	☒	A description of any assumptions or corrections, such as tests of normality and adjustment for multiple comparisons
☐	☒	A full description of the statistical parameters including central tendency (e.g. means) or other basic estimates (e.g. regression coefficient) AND variation (e.g. standard deviation) or associated estimates of uncertainty (e.g. confidence intervals)
☐	☒	For null hypothesis testing, the test statistic (e.g. F, t, r) with confidence intervals, effect sizes, degrees of freedom and P value noted<br>*Give P values as exact values whenever suitable.*
☒	☐	For Bayesian analysis, information on the choice of priors and Markov chain Monte Carlo settings
☒	☐	For hierarchical and complex designs, identification of the appropriate level for tests and full reporting of outcomes
☒	☐	Estimates of effect sizes (e.g. Cohen's *d*, Pearson's *r*), indicating how they were calculated

Our web collection on statistics for biologists contains articles on many of the points above.

Software and code

Policy information about availability of computer code

Data collection	We used R version 3.6.2. We used packages including auk (eBird data processing) and iGraph (network metrics).
Data analysis	We used R version 3.6.2. We used custom written code in addition to packages like iGraph and NetIndices. All code is available on GitHub (DOI: https://doi.org/10.5281/zenodo.4437248) with the exception of the source functions (written by A.B), which can be made available upon request. These functions will be published at a later date.

For manuscripts utilizing custom algorithms or software that are central to the research but not yet described in published literature, software must be made available to editors and reviewers. We strongly encourage code deposition in a community repository (e.g. GitHub). See the Nature Research guidelines for submitting code & software for further information.

Data

Policy information about availability of data

All manuscripts must include a data availability statement. This statement should provide the following information, where applicable:

- Accession codes, unique identifiers, or web links for publicly available datasets
- A list of figures that have associated raw data
- A description of any restrictions on data availability

Hechinger et al. (2011) provides the published, unfiltered data on species interactions. We have included the filtered species and interaction data used in our analyses as Supplementary Data Files 1 and 2. Xiao et al. (2018) provides information on links between functional groups and ecosystem services. Species-level data connecting species and services, as used here, have been submitted as a data paper and is available upon request from the authors (A.K. and L.D.). We used the eBird database and Google Scholar to collect ecosystem service data. All data used to generate the results were available to reviewers.

Field-specific reporting

Please select the one below that is the best fit for your research. If you are not sure, read the appropriate sections before making your selection.

☐ Life sciences ☐ Behavioural & social sciences ☒ Ecological, evolutionary & environmental sciences

For a reference copy of the document with all sections, see nature.com/documents/nr-reporting-summary-flat.pdf

Ecological, evolutionary & environmental sciences study design

All studies must disclose on these points even when the disclosure is negative.

Study description	We simulated 12 sequences of species extinctions on 3 salt marsh food webs that included ecosystem services as nodes in the network. We calculated the robustness of the food web to secondary extinctions and compared that to the robustness of ecosystem services (measuring the extent to which secondary extinctions lead to the loss of ecosystem services in aggregate). We also looked at whether or not individual ecosystem service robustness is associated with the mean trophic level of species providing the service and the redundancy of the services (i.e., the number of species providing a service).
Research sample	We used the food web data from Hechinger et al. 2011. This includes detailed information about species and their trophic interactions for three salt marshes. We used these salt marsh systems because they have overlapping, but discrete species lists and are meant to be replicates. Data was collected with similar methods for each of the salt marshes (in USA and Mexico). These salt marshes served as the basis for ecosystem service assignments. We also used these salt marshes because co-author, L.D., had conducted previous work with 4 ecosystem services at the functional level in these systems.
Sampling strategy	We started with the three salt marsh food webs. To assign ecosystem services, we first conducted a literature synthesis to identify the key ecosystem services associated with salt marshes/estuaries, leading us to a total of 7 ecosystem services. To assign ecosystem services, we conducted another literature review, searching for each unique combination of species-services and any synonyms. We used these three salt marshes and 7 ecosystem services because they are comparable, but discrete. The ecosystem services are provided by overlapping but not exactly similar sets of species, providing variation in structure at both the food web and ecosystem service levels. These numbers are sufficient for the analysis because we ran 12 sequences of species extinctions for all three food webs and their services, providing a sample size of n=36.
Data collection	We collected data on which species provide 7 ecosystem services in the salt marsh food webs. To do this, we (Keyes and Dee) conducted a literature review in Google Scholar, and Keyes analyzed data from eBird using the R package auk.
Timing and spatial scale	We used existing food web data for three salt marshes on the western coast of the United States and Mexico (Hechinger et al. 2011).
Data exclusions	We did not exclude data from the analysis. We did conduct multiple sensitivity analyses, detailed in the Supplemental Information.
Reproducibility	Our study was completed using models and is reproducible given the code and network data we assembled. We did not replicate the experiments multiple times, however we simulated 12 extinction sequences on 3 salt marshes to compare food web and ecosystem service robustness a total of 36 times.
Randomization	We included multiple random sequences of species losses in our models. Specifically, we simulated 1000 random removals of all species, and 100 each of the ecosystem service providers and supporting species.
Blinding	Blinding was not relevant to our study because we used existing food web data.

Did the study involve field work? ☐ Yes ☒ No

Reporting for specific materials, systems and methods

We require information from authors about some types of materials, experimental systems and methods used in many studies. Here, indicate whether each material, system or method listed is relevant to your study. If you are not sure if a list item applies to your research, read the appropriate section before selecting a response.

Materials & experimental systems

n/a	Involved in the study	
☒	☐	Antibodies
☒	☐	Eukaryotic cell lines
☒	☐	Palaeontology and archaeology
☒	☐	Animals and other organisms
☒	☐	Human research participants
☒	☐	Clinical data
☒	☐	Dual use research of concern

Methods

n/a	Involved in the study	
☒	☐	ChIP-seq
☒	☐	Flow cytometry
☒	☐	MRI-based neuroimaging
